# The Interplay of Child Sleep Disturbances, Maternal Burnout, and Marital Quality in Iranian Mothers

**DOI:** 10.1055/s-0046-1829072

**Published:** 2026-10-09

**Authors:** Amir Ali Mazandarani, Masoomeh Karimi

**Affiliations:** 1 Division of Psychology, Department of Behavioral Sciences, The Institute for Research and Development in the Humanities (SAMT), Tehran, Iran; 2 Department of Psychology, Islamic Azad University, Tehran, Iran

**Keywords:** child sleep problems, maternal burnout, marital quality, structural equation modeling, family dynamics

## Abstract

This study examined the relationships between child sleep disturbances, maternal burnout, and marital quality among Iranian mothers. Using a cross-sectional design, data were collected from 564 married mothers of preschool-aged children (aged 5–6 years) through validated questionnaires: the *Children's Sleep Habits Questionnaire* , the *Parental Burnout Assessment* , and the *Quality of Marriage Index* . A structural equation model (SEM) was used to test hypothesized associations among the variables. The final model demonstrated excellent fit to the data. Results indicated a significant positive correlation between child sleep disturbances and maternal burnout ( *r* = 0.374, *p* < 0.001) and significant negative correlations between maternal burnout and marital quality ( *r* = –0.368, *p* < 0.001) and between child sleep disturbances and marital quality ( *r* = –0.217, *p* < 0.001). In the measurement model, strong indicators were identified for each latent variable, including night wakings (β = 0.683) and parasomnias (β = 0.533) for child sleep disturbances, and contrast with the previous maternal self (β = 0.909) and emotional exhaustion (β = 0.864) for maternal burnout. These findings advance theoretical understanding by highlighting key interconnections between child sleep, maternal well-being, and relational outcomes within a specific cultural context. The results underscore the importance of a systemic approach in examining how child sleep disturbances and maternal burnout jointly relate to marital quality, with implications for family well-being interventions.

## Introduction

The quality of marital relationships affects millions of families worldwide, with cascading effects on parenting practices and child development outcomes. 1 Conceptually, marital quality encompasses a global evaluation of the marriage reflecting an overall evaluative perception of the relationship — a preference for and positive appraisal of the union. 2 From a measurement perspective, this global evaluation is captured by integrating key dimensions of relationship health, such as subjective satisfaction, perceived stability, and the overall sense of happiness within the union. 2 High marital quality is important for individual well-being, as it has been linked to better mental and physical health outcomes. 3 4 Moreover, it significantly influences parenting practices, with positive marital relationships fostering effective parenting behaviors. This, in turn, contributes to healthier parent–child relationships and promotes optimal child development. 5 Therefore, maintaining high marital quality is essential for creating a stable and nurturing family environment that supports the well-being of all family members.

Sleep problems are prevalent among preschool-aged children, with studies indicating that approximately 25% experience difficulties such as trouble falling asleep and frequent night awakenings. 6 These disruptions can extend beyond the child, influencing parental well-being and family relationships. 7 8 The spillover hypothesis 9 and family systems theory 7 provide valuable frameworks for understanding the complex interplay between stressors like child sleep problems and marital harmony. These theories suggest that child sleep difficulties can be associated with increased parental fatigue, reduced patience, and amplified conflicts, which may be linked to lower marital satisfaction. 10 11 The cumulative stress of managing child sleep issues may influence both partners' perceptions of relationship quality. 10 Beyond the impact on parents, research supports a bidirectional relationship, indicating that the emotional climate within the marriage—characterized by instability or conflict—acts as a chronic stressor that can disrupt child regulatory processes. 12 Specifically, marital tension may increase a child's physiological arousal and emotional dysregulation, which in turn impairs their ability to initiate or maintain sleep. 12 13 This interconnectedness between child sleep issues and marital quality underscores the importance of examining their associations within a systemic framework that accounts for these complex, reciprocal dynamics. 8 14

Factors associated with the interrelations between child sleep problems and marital quality are crucial for understanding family dynamics. Research has shown that various aspects of the family system, such as parental fatigue and stress, 15 16 along with parenting practices and co-parenting dynamics, 17 are closely linked to these relationships. In collectivistic cultures such as Iran, family dynamics are characterized by highly interconnected boundaries, where individual stressors—such as a child's sleep disturbance—are more likely to permeate the entire family system. 18 Traditional socio-cultural expectations in this context often emphasize the mother as the primary caregiver, placing a significant burden on her to manage child-rearing challenges. 19 Such high expectations for maternal dedication and self-sacrifice can intensify maternal burnout. 20 Maternal burnout, defined as a state of chronic parental stress, 21 has been associated with increased spousal conflict and decreased family relationship quality. 22 By exploring these associated factors, researchers can better understand the pathways through which child sleep problems may be connected to family well-being.

Managing a child's sleep issues may lead to maternal exhaustion 23 and a diminished capacity to cope with daily stressors. 24 Research indicates that child sleep disturbances are strongly associated with increased maternal stress and depressive symptoms. 16 For example, maternal postpartum depression symptoms correlate with more frequent night awakenings and shorter nocturnal sleep duration in infants. 25 Additionally, maternal mental health symptoms have been linked to child sleep behaviors, suggesting a bidirectional relationship between child sleep and maternal well-being. 26 Thus, these findings underscore the critical relationship between child sleep problems and maternal psychological functioning.

The potential link between child sleep challenges and parental burnout has been largely neglected in the research literature. Parental burnout—a state characterized by overwhelming exhaustion related to the parental role, emotional distancing from one's children, and a sense of ineffectiveness in parenting 27 —appears to be an under-researched factor associated with child sleep challenges. Notably, higher levels of maternal burnout have been observed among parents of children with complex needs, 28 indicating that child-related challenges, such as sleep disturbances, may be associated with increased burnout rates. Therefore, investigating the relationship between child sleep problems and maternal burnout is a crucial step toward a more comprehensive understanding of family well-being.

The association between maternal mental health and family well-being is also observed in the context of marital relationships. Conditions such as anxiety, depression, and burnout can diminish a mother's emotional availability, hinder effective communication, and impair conflict resolution within the marriage. 3 Research indicates that maternal depression correlates with decreased marital satisfaction, with higher levels of depressive symptoms linked to lower marital quality. 29 Similarly, maternal burnout has been associated with reduced marital satisfaction, suggesting that the exhaustion and emotional depletion characteristic of burnout adversely affect relationship dynamics. 30 These mental health challenges can create a ripple effect, indirectly impacting the entire family system. 31

Despite established connections among child sleep disturbances, maternal mental health, and marital quality, a significant gap remains in the literature regarding their complex, concurrent associations. While previous studies have examined dyadic relationships between these variables, there is limited research integrating child sleep, maternal burnout, and marital quality within a single structural framework. Examining these dynamics in the Iranian context—where family systems are characterized by strong interpersonal ties and high expectations for maternal caregiving—offers a valuable opportunity to investigate these associations within a collectivistic framework. Such research can provide insights that complement existing evidence from Western contexts. Therefore, the present study aims to extend previous research by comprehensively examining the structural interrelations among child sleep problems, maternal burnout, and marital quality. Specifically, we seek to investigate the pattern of associations between these three factors to understand how they concurrently relate to one another within the family system. These findings are important for advancing a family systems perspective and may inform the development of more effective, family-centered interventions that address the multiple facets of maternal and child well-being.

## Methods

### Participants and Procedure

This cross-sectional study initially recruited 584 married mothers in Islamshahr, Iran. Data were collected online via the Porsline platform. To ensure a high response rate and data quality, preschool managers facilitated the process by distributing the link directly to mothers and providing initial instructions; only mothers who expressed willingness to participate proceeded to the survey. Inclusion criteria were: (a) married mother of a 5–6-year-old child, (b) child attending a private preschool in Islamshahr, and (c) co-residence with the child. The number of children in the household was not an exclusion criterion, and mothers were eligible regardless of their total number of children. Following data screening, 20 participants were excluded due to incomplete questionnaire data (attrition rate = 3.4%), resulting in a final sample of 564 mothers. The study was approved by the Ethics Committee of The Institute for Research and Development in the Humanities (SAMT) and adhered to the 1964 Helsinki Declaration principles. Mothers were informed of the study's objectives, voluntary participation, confidentiality, and right to withdraw. Questionnaire submission indicated informed consent.

### Measures

#### Children's Sleep Habits Questionnaire (CSHQ)

The CSHQ, a parent-reported tool, assesses sleep behaviors in children aged 4–12 years. 32 It includes 33 scored items rated on a 3-point Likert scale (“usually” [5–7 times/week], “sometimes” [2–4 times/week], “rarely” [0–1 time/week]), covering eight subscales: Bedtime Resistance, Sleep Onset Delay, Sleep Duration, Sleep Anxiety, Night Wakings, Parasomnias, Sleep-Disordered Breathing, and Daytime Sleepiness. A Total Sleep Disturbance Score (range: 33-99) is calculated, with higher scores indicating greater sleep problems. Parents also noted whether behaviors were problematic (“yes,” “no,” or “not applicable”). The Persian CSHQ has good reliability (Cronbach's α = 0.80) and validity. 33 In this study, it showed acceptable internal consistency (Cronbach's α = 0.772).

#### Parental Burnout Assessment (PBA)

The PBA measures maternal burnout 34 with 23 items on a 7-point scale (0 = never, 6 = every day), across four subscales: Emotional Exhaustion, Emotional Distancing, Feelings of Being Fed Up, and Contrast with Previous Maternal Self. Higher global scores indicate greater burnout. The Persian PBA has high reliability (Cronbach's α = 0.93) and good validity. 35 In this study, reliability was excellent (Cronbach's α = 0.93).

#### Quality of Marriage Index (QMI)

The QMI assesses marital satisfaction with six items 2 : five on a 7-point Likert scale (1 = strongly disagree, 7 = strongly agree) and one on a 10-point scale (1 = very unhappy, 10 = perfectly happy). Total scores range from 6–42, with higher scores indicating greater satisfaction. The Persian QMI has strong reliability (Cronbach's α = 0.922) and validity. 36 In this study, it showed excellent reliability (Cronbach's α = 0.94).

### Statistical Analysis

Descriptive statistics and Pearson correlations were calculated in IBM SPSS Statistics (Version 27) to summarize sample characteristics and examine bivariate associations among child sleep variables, maternal burnout, and marital quality. Data were screened for missingness, outliers, and accuracy. Normality was evaluated using skewness/kurtosis (|skew| < 2, |kurtosis| < 7), histograms, and Shapiro–Wilk tests; linearity, homoscedasticity, and multicollinearity were checked via scatterplots, residual analyses, and VIF (< 5).

Structural equation modeling (SEM) was conducted in IBM SPSS Amos (Version 24) with Maximum Likelihood estimation. Multivariate normality was assessed with Mardia's coefficient, and multivariate outliers were identified using Mahalanobis distance ( *p* < 0.001). Child Sleep Disturbances and Maternal Burnout were modeled as latent variables, measured by six CSHQ and four PBA subscales, respectively; Marital Quality was modeled as an observed variable. Model fit was evaluated using χ ^2^ /df (< 5.0), RMSEA (< 0.08, 90% CI), CFI (> 0.90), TLI (> 0.90), and parsimony indices (AIC, ECVI). Bootstrapping with 2,000 resamples generated bias-corrected confidence intervals for parameter estimates.

Given the cross-sectional design, mediation analyses were avoided to prevent unsupported causal claims. 37 SEM was instead applied in a correlational framework to estimate the strength and pattern of associations while accounting for measurement error. Statistical significance was set at *p* < 0.05 (two-tailed).

## Results

### Participants' Characteristics

The study involved 564 married mothers from Islamshahr, Iran, with children aged 5–6 years (mean = 5.46, SD = 0.499) attending private preschools. Children were 48.76% boys and 51.24% girls, with 63.83% firstborns. Mothers' mean age was 35.05 years (SD = 5.81); 83% held undergraduate or higher degrees. Employment status included 69.33% at-home/students/unemployed, 20.21% part-time, and 10.46% full-time ( Table 1 ).

**Table 1:** Characteristics of Participants and Their Mothers ( *N* = 564)

Category	Subcategory	Frequency (n)	Percentage (%)	Mean (SD)
A. Child Characteristics				
Gender	Boys	275	48.76	
	Girls	289	51.24	
Age (years)				5.46 (0.499)
Birth Order	Firstborn	360	63.83	
	Second-born	129	22.87	
	Third-born or later	75	13.30	
B. Mother Characteristics				
Education Level	Post-graduate	196	34.75	
	Undergraduate	274	48.58	
	High school	75	13.30	
	Less than high school	19	3.37	
Employment Status	At-home/student/unemployed	391	69.33	
	Part-time	114	20.21	
	Full-time	59	10.46	
Age (years)				35.05 (5.81)

### Variable Description

Total Sleep Disturbances strongly correlated with Bedtime Resistance (r = 0.754, p < 0.01) and Sleep Anxiety (r = 0.734, p < 0.01). Perceived Child Sleep Problem correlated with Total Sleep Disturbances (r = 0.472, *p* < .01) and Maternal Burnout subscales (e.g., Exhaustion: r = 0.305, *p* < .01; Overall Burnout: r = 0.350, *p* < .01). Total Sleep Disturbances also linked to Maternal Burnout (e.g., Overall Burnout, r = .302, *p* < .01). Quality of Marriage negatively correlated with Perceived Child Sleep Problem (r = - 0.187, *p* < 0.01), Total Sleep Disturbances (r = - 0.187, *p* < 0.01), and Overall Maternal Burnout (r = - 0.351, *p* < 0.01) ( Table 2 ).

**Table 2:** Correlations among Child Sleep Variables, Maternal Burnout, and Quality of Marriage

	M (SD)	1	2	3	4	5	6	7	8	9	10	11	12	13	14	15
A. Child Sleep Variables																
1. Bedtime Resistance	11.34 (2.95)	1														
2. Sleep Onset Delay	1.30 (0.56)	0.101*														
3. Sleep Duration	3.90 (1.23)	0.154**	0.266**													
4. Sleep Anxiety	7.85 (2.11)	0.719**	0.053	0.089*												
5. Night Wakings	4.37 (1.39)	0.326**	0.041	0.102*	0.340**											
6. Parasomnias	8.45 (1.72)	0.234**	-0.012	0.161**	0.266**	0.385**										
7. Sleep Disordered Breathing	3.30 (0.81)	0.071	0.002	0.093*	0.092*	0.218**	0.402**									
8. Daytime Sleepiness	9.88 (2.80)	0.165**	0.076	0.185**	0.198**	0.200**	0.215**	0.231**								
9. Total Sleep Disturbances	50.38 (8.08)	0.754**	0.191**	0.375**	0.734**	0.571**	0.572**	0.367**	0.595**							
10. Perceived Child Sleep Problem	4.83 (5.18)	0.376**	0.108*	0.258**	0.385**	0.215**	0.272**	0.169**	0.220**	0.472**						
B. Maternal Well-Being Variables																
11. Exhaustion in Parental Role	11.02 (9.80)	0.174**	0.081	0.125**	0.171**	0.195**	0.198**	0.081	0.204**	0.287**	0.305**					
12. Contrast in Parental Self	4.84 (9.98)	0.187**	0.100*	0.159**	0.162**	0.186**	0.144**	0.027	0.191**	0.273**	0.356**	0.785**				
13. Feelings of Being Fed Up	2.15 (4.47)	0.146**	0.065	0.081	0.122**	0.173**	0.137**	0.066	0.143**	0.217**	0.273**	0.726**	0.748**			
14. Emotional Distancing	2.39 (3.46)	0.197**	0.049	0.121**	0.165**	0.114**	0.172**	0.051	0.215**	0.273**	0.290**	0.585**	0.655**	0.576**		
15. Overall Maternal Burnout	20.40 (22.0)	0.198**	0.089*	0.142**	0.178**	0.199**	0.189**	0.066	0.215**	0.302**	0.350**	0.935**	0.923**	0.855**	0.743**	
16. Quality of Marriage	34.31 (7.98)	-0.162**	-0.060	−0.022	−0.089*	−0.110**	−0.108**	−0.113**	−0.127**	−0.187**	−0.187**	−0.317**	−0.326**	−0.321**	−0.261**	−0.351**

Note. * *p* < 0.05.

** *p* < 0.01.

### Structural Equation Modeling Analysis

SEM examined relationships among Child Sleep Disturbances, Maternal Burnout, and Marital Quality, using 23 variables (11 observed, 12 latent) and 41 parameters. Model fit was excellent: χ ^237^ = 56.15, *p* = 0.023; χ ^2^ /df = 1.52; RMSEA = 0.030; CFI = 0.992; TLI = 0.988. Bootstrapping (2,000 resamples) confirmed parameter stability ( Fig. 1 ).

**Fig. 1.**
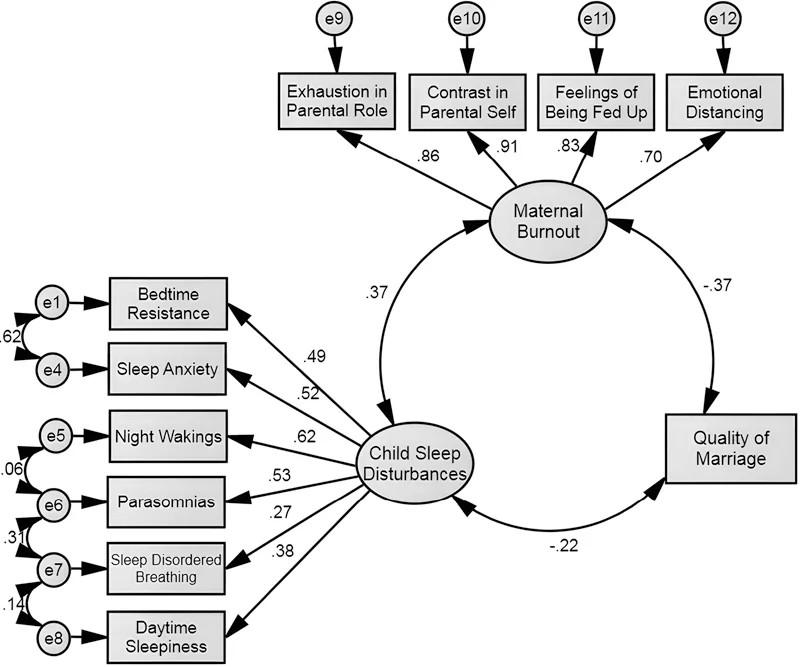
Structural equation model depicting the interrelationship among maternal burnout, child sleep disturbances, and marital quality.

Child Sleep Disturbances was indicated by six CSHQ subscales: Night Wakings (β = 0.683), Parasomnias (β = 0.533), Sleep Anxiety (β = 0.491), Bedtime Resistance (β = 0.463), Daytime Sleepiness (β = 0.434), and Sleep-Disordered Breathing (β = 0.267). Maternal Burnout was measured by four PBA subscales: Contrast with Previous Maternal Self (β = 0.909), Emotional Exhaustion (β = 0.864), Feelings of Being Fed Up (β = 0.828), and Emotional Distancing (β = 0.703). All loadings were significant ( *p* < 0.001).

Child Sleep Disturbances positively correlated with Maternal Burnout (r = 0.374, *p* < 0.001), while Marital Quality negatively correlated with both (r = − 0.217 and −0.368, *p* < 0.001). Error covariances (e.g., Bedtime Resistance ↔ Sleep Anxiety, r = 0.623, *p* < 0.001) improved model fit. SMCs showed high variance explained for Maternal Burnout (0.494–0.827) but lower for Child Sleep Disturbances (0.071–0.466). Sleep Onset Delay and Sleep Duration were excluded due to low loadings. Night Wakings and Parasomnias were key for Child Sleep Disturbances; Contrast with Previous Maternal Self and Emotional Exhaustion were prominent for Maternal Burnout.

## Discussion

This study investigated the correlational relationships among child sleep disturbances, maternal burnout, and marital quality. The structural equation model revealed significant positive and negative covariances among all three variables, demonstrating a complex interplay. For child sleep disturbances, Night Wakings and Parasomnias were the strongest indicators of the latent construct, while for maternal burnout, Contrast with the Previous Maternal Self and Emotional Exhaustion were the primary markers. The analysis showed that child sleep disturbances were positively correlated with maternal burnout, and both were negatively correlated with marital quality. These results highlight the intricate interrelations between child sleep issues, maternal well-being, and family dynamics. Importantly, the present findings provide evidence for an interconnected network of associations where maternal burnout serves as a central point of intersection, reflecting the personal toll of managing child sleep problems while concurrently being associated with the health of the marital relationship. The present study also acknowledges that these relationships are likely bidirectional. 8

From the individual perspective of the mother, the correlational findings highlight the significant personal strain potentially associated with caregiving responsibilities. Persistent child sleep disturbances are often accompanied by sleep deprivation for mothers, a phenomenon well-documented in prior research. 23 38 This physical exhaustion is often observed alongside heightened anxiety about the child's health and developmental outcomes, further compounding maternal distress. 39 40 Over time, these pressures are associated with burnout, characterized by emotional depletion, physical fatigue, and a diminished capacity to manage daily tasks. 15 41 Theories of stress and coping posit that such chronic demands may be linked to the erosion of psychological resources, leaving mothers vulnerable to strain. 42 In the Iranian context, where societal expectations often emphasize maternal self-sacrifice and primary caregiving roles, 43 this depletion may be particularly acute, potentially amplifying the emotional toll on mothers. Consequently, this individual experience of burnout appears to be a significant factor shaping broader family interactions.

From a family systems perspective, maternal burnout is a key factor in the interconnectedness of child sleep problems with the entire household. The disruption of nightly routines due to a child's sleep difficulties is associated with elevated stress levels, particularly for the mother, who typically assumes the majority of caregiving duties. 44 45 This stress, when prolonged, is related to burnout, which then appears to be linked to the parental relationship as both partners contend with the emotional and practical consequences. 46 47 The resulting fatigue and irritability can impair mutual support and communication, potentially contributing to tension throughout the family unit. 48 Family systems theory underscores this interconnectedness, suggesting that stress in one subsystem—such as the mother-child relationship—is often associated with strain in others, including the marital dyad. 9 31 Research corroborates that child sleep disturbances are linked to broader family challenges, including increased conflict and reduced relational satisfaction. 11 Thus, these sleep-related stressors are an important component of the relational strain observed across the family system.

## Conclusion

In conclusion, the present study provides a more nuanced understanding of family dynamics by illustrating that maternal burnout is closely associated with the relationship between child sleep disturbances and marital quality. It illustrates how individual stressors, such as disrupted sleep, may be related to relational outcomes, aligning with holistic perspectives on family functioning. On a practical level, these findings suggest the importance of interventions that simultaneously target child sleep problems and maternal well-being to support marital harmony. For example, behavioral sleep training has been shown to improve child sleep patterns while alleviating maternal stress, 49 which may in turn be associated with reduced familial tension. Additionally, family-oriented approaches—such as couple counseling to enhance communication or respite care to lighten the maternal load—could be part of a strategy to address the cycle of stress and relational discord. 50 These insights highlight the value of early screening for both sleep difficulties and burnout as a potential strategy to mitigate long-term family strain.

Despite its contributions, this study has limitations that merit attention. A primary limitation stems from the cross-sectional design, which prevents causal inferences and precludes a definitive interpretation of the directionality of the observed relationships. The use of a correlational structural model was a deliberate choice to align with family systems theory, which posits that all elements within a family are interconnected and bi-directionally related, making it difficult to designate one factor as entirely antecedent to another. However, this approach inherently limits the ability to draw conclusions about temporal causality. Longitudinal studies are therefore essential to clarify the developmental and temporal dynamics of these associations.

Furthermore, the focus on Iranian mothers limits the generalizability of the findings, necessitating replication across diverse cultural settings where parenting norms and family structures may differ. Importantly, the present study did not account for household size or the availability of extended family support (e.g., assistance from grandparents or other co-residing relatives), which are common in the Iranian context and could potentially buffer the impact of child sleep disturbances on maternal well-being. Additionally, while the birth order of the child was recorded, the total number of children in each family was not included as a covariate in the analysis, which may influence the level of parental burnout. The reliance on self-reported and parent-reported data introduces potential biases, which could be mitigated in future research by incorporating objective measures like actigraphy to assess sleep and physiological markers of stress. Additionally, exploring paternal burnout and its associations alongside maternal burnout could significantly enrich our understanding of how these factors operate within the entire family system. Addressing these gaps will strengthen the evidence base for the development of more comprehensive and effective family-centered interventions.
